# The Soft- and Hard-Heartedness of Cardiac Fibroblasts: Mechanotransduction Signaling Pathways in Fibrosis of the Heart

**DOI:** 10.3390/jcm6050053

**Published:** 2017-05-19

**Authors:** Kate M. Herum, Ida G. Lunde, Andrew D. McCulloch, Geir Christensen

**Affiliations:** 1 Institute for Experimental Medical Research, Oslo University Hospital and University of Oslo, 0450 Oslo, Norway; i.g.lunde@medisin.uio.no (I.G.L.); geir.christensen@medisin.uio.no (G.C.); 2 Center for Heart Failure Research, Oslo University Hospital, 0450 Oslo, Norway; 3 Department of Bioengineering, University of California San Diego, La Jolla, CA 92093, USA; amcculloch@ucsd.edu; 4 Department of Medicine, University of California San Diego, La Jolla, CA 92093, USA

**Keywords:** fibrosis, cardiac fibroblast, myofibroblast, mechanotransduction, stiffness, extracellular matrix, integrins, syndecan, cytoskeleton, linker of the nucleoskeleton and cytoskeleton

## Abstract

Cardiac fibrosis, the excessive accumulation of extracellular matrix (ECM), remains an unresolved problem in most forms of heart disease. In order to be successful in preventing, attenuating or reversing cardiac fibrosis, it is essential to understand the processes leading to ECM production and accumulation. Cardiac fibroblasts are the main producers of cardiac ECM, and harbor great phenotypic plasticity. They are activated by the disease-associated changes in mechanical properties of the heart, including stretch and increased tissue stiffness. Despite much remaining unknown, an interesting body of evidence exists on how mechanical forces are translated into transcriptional responses important for determination of fibroblast phenotype and production of ECM constituents. Such mechanotransduction can occur at multiple cellular locations including the plasma membrane, cytoskeleton and nucleus. Moreover, the ECM functions as a reservoir of pro-fibrotic signaling molecules that can be released upon mechanical stress. We here review the current status of knowledge of mechanotransduction signaling pathways in cardiac fibroblasts that culminate in pro-fibrotic gene expression.

## 1. Cardiac Fibrosis and Heart Disease

Cardiac fibrosis, the excessive accumulation of extracellular matrix (ECM), occurs in nearly all types of heart disease including myocardial infarction, aortic stenosis, dilated cardiomyopathy, diabetic cardiomyopathy and hypertrophic cardiomyopathy [1,2,3,4,5,6]. It is characterized by dysregulated production, post-translational modification, enzymatic processing and turnover of collagens (mainly type I and III in the heart) and other ECM components such as proteoglycans [2,7,8,9].

Cardiac fibrosis comes in several forms, i.e., perivascular, interstitial and focal, and this ECM remodeling increases the risk for arrhythmias and may reduce pumping function as in heart failure with reduced ejection fraction (HFrEF). Cardiac fibrosis is also a main driver of heart failure with preserved ejection fraction (HFpEF) as it increases myocardial stiffness, thereby compromising the distensibility of the ventricles and impairing the filling capacity of the heart [10].

There is to date no effective treatment for cardiac fibrosis. This could, in part, be due to the poor understanding of the function of the cell type responsible for ECM production, the cardiac fibroblast [11,12,13,14,15]. Currently, the cardiac ECM and fibroblast pathophysiology are under intense investigation [16]. The ECM is accepted as a dynamic and active player in health and disease and a central role for cardiac fibroblasts in development of fibrosis has been established [9]. Hopefully, this expanding effort and accumulation of knowledge will lead to the discovery of novel targets and anti-fibrotic therapies.

In order to be successful in preventing, attenuating or reversing cardiac fibrosis, it is essential to understand the processes leading to ECM production and accumulation [2]. Altered mechanical properties of the heart occurs early in many types of cardiac disease [17]. Thus, in addition to increased neurohormonal activity [18,19] and sterile inflammation [20,21] that are well-known to increase ECM production, mechanical factors are crucial for the development of fibrosis. How these mechanical cues are translated into cardiac fibroblast responses is not completely understood. We here review the current state of knowledge on pro-fibrotic mechanotransduction signaling pathways in cardiac fibroblasts.

## 2. Mechanical Forces of the Heart

Cells in the heart are continuously subjected to different types of mechanical forces, and changes in these mechanical forces during cardiac disease induce responses in cardiac fibroblasts. Stress and strain in the heart wall are three-dimensional and non-uniform. In addition to normal tensile (pulling) and compressive (pushing) components, parallel and transverse to the planes of the wall, there is significant shearing (friction) during filling and ejection, as evidenced by the torsional deformations seen during the cardiac cycle. An overview of the mechanical terms used to describe the various mechanical forces of the heart is presented in Box 1.

Since stresses represent the three-dimensional forces of interaction within the tissue, they cannot be measured directly, mostly because any device or implant intended to measure tissue stresses inevitably affects them. Therefore, wall stress distributions are estimated with the aid of computational models [22] that solve Newton’s force balance equations for the myocardium. The models make use of knowledge of the three-dimensional mechanical properties of the muscle at rest and during contraction, and the known external loads acting on the heart walls, namely the chamber and pericardial pressures.

On the other hand, strains represent three-dimensional regional shape (i.e., length) changes in the tissue such as systolic fiber shortening and wall thickening. Their regional distributions can be measured non-invasively in humans and animal models using techniques such as speckle tracking echocardiography [23] or tagged magnetic resonance imaging (MRI) [24], both of which rely on imaging the motion of material points in the walls. By comparing measured three-dimensional strains with models, their computations of wall stress can be reliably validated. However, it remains challenging to relate macroscopic wall stresses and strains with cell scale mechanics. For example, how stresses are distributed between myofilaments and the ECM can still be challenging to discriminate definitively.

To understand the direct effect of force on cardiac fibroblast phenotype, in vitro systems have been developed. These include measuring passive and active tensile forces of intact myocardial strips, and applying strain to cells by static or cyclic stretching of cardiac cell cultures. These methods have been extensively reviewed elsewhere [25]. More recently, the effect of matrix stiffness on cardiac fibroblast phenotype has received great attention as fibroblasts harbor great plasticity and alter their phenotype in response to tissue and ECM stiffness [25,26,27,28,29]. Stiffness is measured in Pascal (Pa), describing the material’s Young’s elastic modulus. By the use of atomic force microscopy, stiffness has been estimated to be 10 kPa for the healthy myocardium and 20–100 kPa for the fibrotic myocardium [30,31], whereas standard culturing conditions have stiffness in the GPa (10^9^ Pa) range. Hence, standard in vitro cultures of cardiac fibroblasts are, in most cases, representative of myofibroblasts. To maintain a quiescent cardiac fibroblast phenotype in vitro, cells must be cultured on soft hydrogels where the stiffness can be tuned to better mimic the in vivo mechanical environment. These include polyacrylamide gels [32,33,34] and hyaluronic acid gels that can be stiffened or softened during cell culturing [35,36,37]. However, few studies have to date combined soft hydrogels with the application of stretch [25,27,38,39]. Thus, much is yet to be learned about the stretch-induced responses of quiescent cardiac fibroblasts and how each of the mechanical cues observed in the intact heart are translated into biological alterations in fibroblast function and fibrosis.

Box 1 **Mechanical forces of the heart.**

**Strain** refers to the deformation of a material. Strain is positive if the material fibers are stretched and negative if they are compressed.

**Stress** is force per unit area.

**Tensile forces** are pulling forces that act to lengthen a material in the direction of the applied force.

**Tensile stress** is the tensile force per unit area. Tensile stress applied *to* a material gives rise to **tensile strain,** which measures the lengthening (i.e., **stretch**) of a material. Tensile stress developed by a cell (due to myofilament or cytoskeletal contraction) will tend to cause compressive strain or shortening along the axis of tension development. During ventricular filling, acute diastolic tensile stress and strain is applied to the myocardium, while chronic left ventricular pressure overload will increase the tensile stress in the myocardium during systole.

**Stiffness** is the extent to which a material resists deformation in response to an applied force, and is the inverse of **compliance**. Extracellular matrix (ECM) stiffness is mainly determined by fibrillar collagens (collagen type I and III in the heart) and the degree of collagen cross-linking [40,41,42]. Cell stiffness is largely determined by prestress [43].

**Prestress** is the stress remaining in the cell or tissue when external forces on the tissue are removed. Cellular prestress is often attributed to tension in stress fibers of the cytoskeleton balanced by compression in microtubules and/or cytoplasmic pressure (positive pressure is a negative, compressive stress). In tissue or cells adhered to ECM, these pulling forces are resisted by external tethers to the ECM. Prestress is modulated by cell stiffness [43] and essential for cell differentiation [44].

**Cell traction force** is the tension exerted on the ECM and other cells via adhesion receptors (integrins and cadherins) as a result of cell shortening of the contracting cytoskeleton. It can be computed from measurements of the strains induced by cell contraction.

**Compressive force** is a pushing force; External forces that act to shorten the material in the direction of the applied force are compressive.

**Shear tractions** are tangential forces per unit area such as those due to friction or forces applied by a cell adhered to a matrix surface. The torsional rotation of the ventricle during filling and ejection creates shearing stresses caused by the helical arrangement of myofibers in the wall. Transverse shearing stresses and strains also exist between myocardial laminar “sheets” during systole [45]. In vascular biology, **shear stress** frequently refers to the forces of fluid flow on the surface of cells e.g., due to blood flow. These shear stresses tend to be orders of magnitude lower than shear stresses due to cell interactions with solid matrix but are still biologically significant.

## 3. Cardiac Fibroblasts and Mechanotransduction

**Figure 1:**
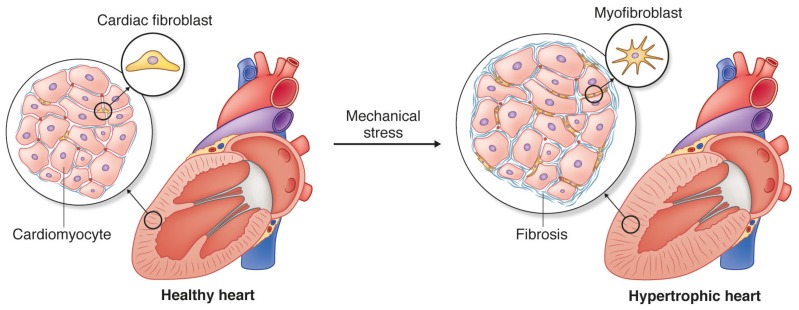
Cardiac fibroblasts are located in between cardiomyocytes where they ensure the appropriate amount and composition of extracellular matrix (ECM) in the healthy heart. Mechanical stress induces fibrosis during cardiac remodeling, e.g., hypertrophic remodeling. Fibrosis compromises cardiac function, and results from activation of cardiac fibroblasts and transition into a myofibroblast phenotype characterized by excessive production of ECM.

Cardiac fibroblasts are essential in health and disease. In the healthy heart they are rather quiescent, with low ECM producing activity maintaining a baseline turnover of ECM proteins. Biochemical and mechanical cues activate cardiac fibroblasts leading to increased production of ECM [25,46] (Figure 1). This is beneficial in the initial phase after a myocardial infarction or induction of pressure overload of the heart, as it enables adjustment of ECM production to meet the requirements of the changing extracellular environment [47]. As such, upregulation of ECM genes and remodeling of the ECM ensures structural stability to withstand increases in mechanical load and repair the myocardium following injury; e.g., fibrosis is necessary to prevent myocardial rupture following an infarction. However, persistently high ECM-producing activity is associated with myocardial stiffening and impaired function [11,48].

The activated cardiac fibroblast exhibits multiple overlapping phenotypes [47,49] with some common characteristics that distinguish them from quiescent fibroblasts. These include increased proliferation, excessive production of ECM, large and strong focal adhesions, and expression of smooth muscle α-actin (SMA), the contractile actin filament which is part of the normal smooth muscle cell’s contractile cytoskeleton. Incorporation of SMA into stress fibers enables a slow and endured contraction (also called contracture), resulting in rearrangement and remodeling of the ECM. Due to this smooth muscle-like phenotype, fully activated cardiac fibroblasts are referred to as myofibroblasts [46]. Recently, it was demonstrated that cardiac fibroblasts can also adopt alternative cell fates following myocardial injury [50] including an osteoblast-like fate associated with the calcification process accompanying fibrosis [51], underlining the vast plasticity of cardiac fibroblasts.

Many cell types may adopt a myofibroblast phenotype in vitro. Thus, the cellular origin of pro-fibrotic cardiac myofibroblasts in vivo has been debated. The traditional view is that myofibroblasts in the heart derive from resident cardiac fibroblasts and this is supported by lineage tracing studies using the collagen type I alpha 1 chain (collagen1a1) gene promoter controlling the green fluorescent protein (GFP) reporter [52]. This view has been challenged by the finding that endothelial cells [53], bone-marrow-derived circulating progenitor cells [54,55], and pericytes surrounding blood vessels [56] can also adopt a myofibroblast phenotype and hence contribute to the cardiac myofibroblast population. However, recent compelling evidence demonstrate that transcription factor 21 (Tcf21)-expressing tissue-resident fibroblasts indeed give rise to cardiac myofibroblasts and deletion of Tcf21-expressing fibroblasts blunt the development of fibrosis [57]. Thus, it seems that the majority of cardiac myofibroblasts are indeed derived from resident cardiac fibroblasts [58]. 

No specific marker protein exclusively expressed in the myofibroblast has been identified, possibly due to the range of fibroblast-to-myofibroblast phenotypes. Myofibroblasts are most commonly defined by high expression of collagen I, and de novo expression of SMA and the extradomain A (EDA) splice variant of fibronectin [59]. Also, platelet-derived growth factor receptor β (PDGFRβ) [60] and the matricellular protein periostin are de novo expressed in myofibroblasts [57,61].

Mechanical cues are major drivers of cardiac fibroblast activation [49] and act directly on cardiac fibroblasts, or by inducing paracrine signals from mechanically stressed cardiomyocytes [50]. An example of the latter was recently demonstrated by our group showing an increase in cardiac fibroblast proliferation when cultured in the same media as stretched cardiomyocytes [39]. Persistent elevated stretch of cardiac fibroblasts stimulates sustained production of ECM [39,62] causing myocardial stiffening. Interestingly, stretch-induced transcription of certain ECM genes is dependent on matrix stiffness; i.e., cardiac fibroblasts cultured on 3 kPa substrates display different stretch responses than cardiac fibroblasts on 8 kPa substrates [39]. Although the stiffer myocardium will be more resistant to stretch, the mechanical force that arises with the increased stiffness will itself support continued activity of myofibroblasts [39]. As such, the cellular component of focal fibrotic regions of the heart is almost exclusively myofibroblasts [63]. A better understanding of how activated cardiac fibroblasts respond to ECM stiffness and stretch will be important for therapeutic targeting of fibrosis [64].

**Figure 2:**
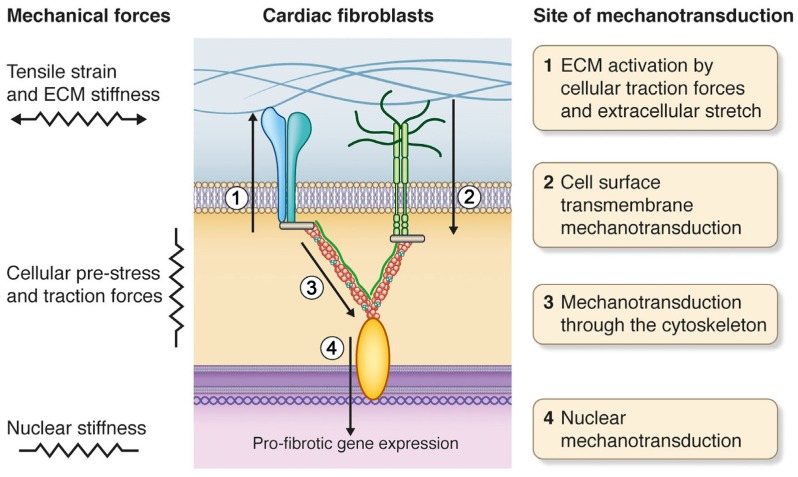
Mechanical forces are translated into biochemical activity by mechanotransduction, which occurs at different cellular sites and by “activation” of the ECM. In cardiac fibroblasts, mechanical stress leads to pro-fibrotic gene expression.

Cells interpret mechanical forces by mechanotransduction, the conversion of mechanical stimuli into chemical activity [65]. Changes in the cell’s mechanical environment cause activation of intracellular signaling pathways that lead to changes in gene expression, cell phenotype and function. Mechanotransduction can occur at multiple cellular locations including the plasma membrane, cytoskeleton and nucleus (Figure 2). Moreover, the ECM can be “activated” by stretch and stiffening [9,66,67]. Due to the great importance of strain and stiffening for pathological ECM remodeling, cell responses to these forces have been the focus of numerous studies and will reviewed here.

## 4. Mechanical Activation of Extracellular Matrix: A Vigilant Pro-Fibrotic Reservoir

Both stretch and stiffness of the ECM will determine how prone the ECM is to “activation”; i.e., the liberation of ECM-derived molecules that stimulate pro-fibrotic cell responses [9,64]. Although not traditionally considered mechanotransduction, compelling evidence describes ECM “activation” by myofibroblasts as a central part of fibrosis development [60,67,68,69,70,71]. ECM stiffness is in constant balance with the cell’s intrinsic stiffness, or pre-stress [43]. However, myofibroblasts gain contractile properties allowing them to “pull” on the ECM. This is termed cell traction forces (Box 1) and has recently been shown to activate ECM-stored signaling molecules in a specific manner [60,68]. There is also evidence suggesting that an excessively stretched ECM will release or expose signaling molecules that are normally concealed by the intact ECM. This may represent a mechanism to alert resident cells of increased mechanical burden [21,72].

### 4.1. Transforming Growth Factor β Activation; A Potent Signal Activated in ECM during Increased Stress

**Figure 3:**
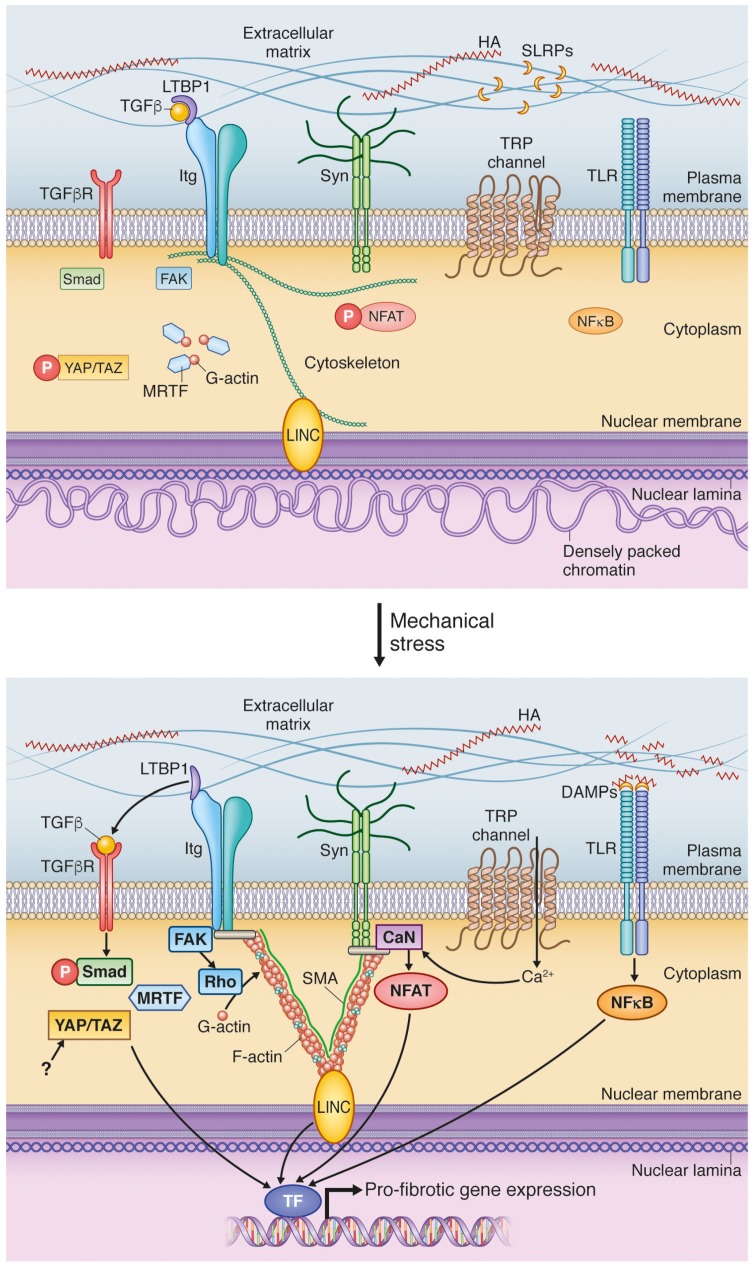
Mechanotransduction signaling pathways in the cardiac fibroblast. Hyaluronic acid (HA), small leucine-rich proteoglycans (SLRPs), latent TGFβ binding protein 1 (LTBP1), transforming growth factor β (TGFβ), TGFβ receptor (TGFβR), integrins (itg), syndecan (syn), transient receptor potential (TRP) channel, toll-like receptor (TLR), focal adhesion kinase (FAK), Yes-associated protein (YAP), transcriptional coactivator with PDZ-binding motif (TAZ), myocardin-related transcription factor (MRTF), nuclear factor of activated T-cells (NFAT), nuclear factor kappa-light-chain-enhancer of activated B cells (NFκB), danger-associated molecular patterns (DAMPs), calcineurin (CaN), smooth muscle α-actin (SMA), linker of nucleoskeleton and cytoskeleton (LINC), transcription factors (TF).

Transforming growth factor β (TGFβ) is the most well-known driver of myofibroblast differentiation [73], inducing pro-fibrotic gene expression through the canonical signaling pathway with activation of Smad2/3, and through non-canonical signaling pathways including activation of Rho-MRTF-A (myocardin-related transcription factor A) [74]. TGFβ is secreted to the extracellular environment in an inactive protein complex consisting of TGFβ and latency-associated propeptide (LAP) which binds to latent TGFβ binding protein 1 (LTBP-1) in the ECM [75,76,77]. The LAP contains an Arg-Gly-Asp (RGD) sequence which is a common ligand for several integrins including αv. As such, all αv integrins are known to bind to RGD in LAP [60,69,70,75,78], and liberate TGFβ from its binding complex in response to cell traction forces (Figure 3) [60,79,80]. This enables storage of TGFβ in the ECM wherefrom it can be rapidly activated upon cellular contraction by myofibroblasts. 

Interestingly, stretch can induce release of active TGFβ from the ECM even in the absence of cells, suggesting that increased ECM stretch will mechanically open the latent complex and activate TGFβ. The amount of stretch necessary to activate TGFβ varies among tissues and depends on the degree of ECM organization, with highly organized ECM having higher bioavailability of TGFβ [81]. Such cell-independent effects of stretch on TGFβ activation have been demonstrated in tendon, a connective tissue characterized by highly organized collagen fibrils with high stretch burden [82]. Accompanying the variations in stiffness among tissues, it seems logic that the mechanical thresholds for TGFβ activation and thus pathological myofibroblast differentiation vary according to the basic stretch level.

Critical regulators of ECM organization are the cross-linking enzymes lysyl oxidase (LOX) and transglutaminase 2 (TG2). LOX and LOX-like (LOXL) enzymes are upregulated in the heart in response to mechanical stress and are associated with fibrosis, diastolic dysfunction and heart failure [40,83,84]. In addition to increasing collagen organization and thus TGFβ bioavailability, LOXL2 was recently found to stimulate cardiac fibroblasts to produce TGFβ, further enhancing TGFβ signaling [84]. Inhibition or genetic disruption of LOXL2 reduced cardiac fibrosis in response to left ventricular pressure overload and improved overall cardiac function [84].

Direct activation of TG2 by cellular traction forces has been demonstrated for vascular smooth muscle cells [85]. The extracellular domain of the transmembrane proteoglycan syndecan-4 has been shown to promote collagen cross-linking through regulation of cell surface TG2 trafficking and activity [86,87,88], and collagen cross-linking by LOX in in vitro [89], suggesting that cells may regulate ECM structure and thus TGFβ bioavailability through inside-out signaling. Although only demonstrated for TGFβ, mechanical activation of ECM-stored growth factors and cytokines may represent a general mechanism for initiation of pro-fibrotic signaling [90,91,92].

### 4.2. DAMPs Are Stress-Induced Initiators of Inflammation and Fibrosis

The ECM may itself activate cardiac fibroblasts in response to mechanical stress by activating toll-like receptors (TLRs; Figure 3). TLRs are central for the innate immune system’s response to pathogen-associated molecular patterns (PAMPs) such as lipopolysaccharide (LPS) derived from the bacterial cell wall. Endogenous molecules that activate these same pathways are called danger-associated molecular patterns (DAMPs) and are associated with sterile inflammation [21]. DAMPs include intracellular cell components such as mitochondrial DNA [93,94], heat-shock proteins [95,96,97], high mobility group box 1 (HMGB1) [98,99] and IL1α [100,101] that are released into the extracellular space during cellular stress, as well as ECM-derived molecules released during tissue damage. The latter group includes tenascin C [102], fibronectin EDA [72], and ECM-localized small leucine-rich proteoglycans (SLRPs) biglycan and decorin [103].

Downstream signaling of TLRs involves activation of the transcription factor nuclear factor kappa-light-chain-enhancer of activated B cells (NFκB). Lumican, also a member of the SLRP family, induces pro-fibrotic signaling through activation of NFκB and is associated with increased cardiac fibrosis and development of heart failure in mice subjected to left ventricular pressure overload [104,105]. Moreover, the shed extracellular domain of the transmembrane proteoglycan syndecan-4 activates NFκB, and affects transcription of collagens and SMA in cultured cardiac fibroblasts [106]. Smaller fragments of the large extracellular carbohydrate chain hyaluronic acid (HA) have also been found to activate TLRs [107,108]. Although there is no direct proof that mechanical forces alone can induce the release of ECM-derived DAMPs, it is plausible that high mechanical load will cause some degree of ECM damage with the consequence of DAMP release. SLRPs, syndecans, glypicans and hyaluronic synthase (HAS) 1 and 2 are upregulated in the pressure overloaded heart [104,105,109,110,111,112] and could represent such DAMPs in the mechanically stressed heart. 

Conceptually, the role of ECM as a reservoir for pro-fibrotic signaling molecules is attractive. The existence of signaling factors that are ready to be released from the ECM by mechanical force would constitute a rapid and efficient mechanism for alarming cardiac fibroblasts of tissue injury or stress. Presumably, the amount of force needed to release DAMPs by mechanical disruption of the ECM is somewhat higher than the force needed for the specific activation of TGFβ. Such a difference in activation threshold would enable responses fine-tuned to the degree of a mechanical burden. 

## 5. Cell Surface Mechanotransduction: A Strained Relationship between the ECM and Cell Interior

Mechanotransduction at the cell surface culminates at focal adhesions, specialized plasma membrane protein complexes comprised of adhesion receptors, signaling molecules and cytoskeletal proteins [113]. Mechanotransduction is mediated through transmembrane adhesion receptors that have the unique ability to sense the extracellular mechanical environment and translate it into cellular responses. They are attached to the ECM as well as the cytoskeleton thereby forming a physical link from the exterior to the interior of the cell. Of these transmembrane adhesion proteins, integrins are the best understood when it comes to mechanotransduction [114] but also other proteins such as transmembrane proteoglycans [109] can transmit mechanical signals across the membrane (Figure 3). Since mechanotransduction can occur in both directions, cell surface signaling through adhesion receptors is often referred to as inside-out and outside-in signaling [115,116]. Mechanotransduction between neighboring cells can occur through cell-cell attachment sites called adherans junctions. This has recently been reviewed for cardiac fibroblasts elsewhere [114], thus we here focus on ECM-cell mediated mechanotransduction. 

### 5.1. Integrins Are Main Constituents of Focal Adhesions Integrating Extracellular and Intracellular Signals

Integrins are adhesion receptors that exist as heterodimers consisting of one α and one β subunit. Upon binding to extracellular ligands, including fibronectin and collagen, integrins cluster to form focal adhesions. In addition to being essential for focal adhesion assembly, integrins are central mediators of mechanotransduction. Integrins interact with 156 known partners collectively referred to as the “integrin adhesome” [117]. Considering this large number of interaction partners, it is no surprise that integrins cross-talk with other membrane receptors and down-stream signaling pathways including G-protein coupled receptors [118] and tyrosine kinase receptors [119,120], affecting adrenergic and growth factor signaling. Comprehensive reviews of integrin cross-talk in the context of fibrosis have been published previously [119,121,122]. Here we discuss the direct down-stream signaling pathways of various integrins that are activated by mechanical cues and associated with fibrosis in the heart.

Cardiac fibroblasts express several of the 24 known integrins, many of which are upregulated during development of cardiac fibrosis [114]. Stretch-induced activation of pro-fibrotic signaling pathways depends on which integrin subunits are enganged [123]. Many cardiac β1 integrins can induce cardiac myofibroblast differentiation in response to mechanical forces [114,124], including collagen receptors α1β1 and α3β1, and fibronectin receptors α5β1 and α8β1, and have been associated with differences observed between soft (healthy) and hard (fibrotic) hearts [68,125,126,127,128,129]. Of the β2 integrins, α1β2 is the main collagen I-binding integrin of the healthy heart and, in contrast to the disease-associated β1 integrins, α1β2 is protective against fibrosis as it regulates collagen I turnover by inducing production of matrix metalloproteinase 1 [130,131,132,133]. Thus, blocking this integrin causes accumulation of collagen and development of fibrosis. β3 integrins bind to RGD sequences of collagen, fibronectin and vitronectin and are necessary for pressure overload-induced myocardial fibrosis [134,135] and have been shown to play a role in mechanical induction of myofibroblast differentiation [136].

The collagen-binding integrin α11β1 is particularly enriched at sites with well-organized interstitial collagen networks [137] and has been associated with fibrosis in several organs including the heart [138,139,140,141,142]. α11 is involved in myofibroblast differentiation [139,141,143], and binds strongly to glycated collagen thereby inducing pro-fibrotic signaling [143]. In vivo, glycated cardiac collagen is associated with a stiffer matrix [42,144], suggesting that α11 may be important for sensing and regulating myocardial stiffness. Indeed, studies in cancer-associated fibroblasts show a close link between presence of α11 and extent of collagen cross-linking [145], an important determinant of ECM stiffness. Furthermore, α11 is associated with left ventricular remodeling in patients with heart failure and genetically modified mice lacking α11 have attenuated diabetes-related cardiac fibrosis [146]. It will be important to pinpoint the mechanisms whereby α11 induces fibrosis in response to mechanical cues.

Since integrins have no intrinsic enzymatic activity, down-stream signaling relies on intracellular interaction with enzymes and adaptor proteins through their cytoplasmic domain [147]. The adaptor protein talin forms a link from integrins to the cytoskeleton and mediates recruitment of signaling molecules to focal adhesions in response to mechanical stimuli [148]. Deletion of talin prevents fibrosis in the pressure-overloaded heart by preventing activation of the signaling molecules mitogen-activated protein kinase (MAPK) p38, extracellular signal-regulated protein kinases 1 and 2 (ERK1/2), protein kinase B (PKB/Akt) and glycogen synthase kinase 3β [149]. Interestingly, it was recently shown that talin is necessary for sensing substrate stiffness [150]. Above a rigidity threshold of 5 kPa, talin structure unfolded thereby recruiting vinculin, promoting focal adhesion formation and inducing cell traction forces and nuclear translocation of the mechanosensitive transcription regulator Yes-associated protein (YAP). These results place talin in the center of mechanotransduction in response to substrate stiffness [150].

Integrins directly and rapidly activate nonreceptor protein tyrosine kinases such as focal adhesion kinase (FAK), Src and Fyn in response to tension and stiffness [39,151,152]. FAK induces a cascade of signaling events involving ERK1/2 and MAPKs [115] which are known to induce myofibroblast differentiation and ECM production [153,154,155]. FAK, Src and Fyn facilitate the activation of Rho GTPases through activating guanine nucleotide exchange factors (GEFs) and GTPases-activating proteins (GAPs) in response to increased tension [156,157]. Rho is important for development of fibrosis demonstrated by the inhibitory effect Rho and Rho kinase blockers on cardiac fibrosis [158]. The importance of FAK in cardiac fibrosis was recently demonstrated in vivo where application of a FAK inhibitor could inhibit myocardial fibrosis following myocardial infarction, although the trigger for FAK activation in that study was suggested to be hypoxia and not mechanical stress per se [159].

Integrins also interact with other adhesion receptors. As such, the ectodomains of syndecans have been found to promote integrin-mediated adhesion of mesenchymal cells, although the mode of interaction is not known [160,161]. The only member of the integrin family known to directly interact with syndecans (syndecan-1 and 4) via its cytoplasmic tail is α6β4 which is not expressed in cardiac fibroblasts [162]. Direct cytoplasmic interaction between cardiac integrins and syndecans have not been identified, although the existence of such interactions is a possibility given their overlapping roles in regulation of fibroblast function.

### 5.2. Syndecan-4 Is Part of the Mechanosensory Apparatus of Fibroblasts

Syndecans comprise a family of four (syndecan-1 to 4). They are transmembrane proteins extracellularly substituted with covalently attached, linear polysaccharide glycosaminoglycan (GAG) chains that bind molecules in the ECM [163]. The core protein of syndecans is relatively small (20–45 kDa). Despite their short cytoplasmic domain, it has become clear that syndecans have important roles in intracellular signaling. Moreover, the binding of syndecan GAG chains to extracellular molecules [164] and of the cytoplasmic domain to the cytoskeleton [165] make syndecans suited for sensing and translating mechanical cues, affecting cells and tissues.

Although all four syndecans are expressed and regulated in heart diseases with increased wall stress [111,166], only for syndecan-4 is there evidence suggesting a role in mechanotransduction in the myocardium. Syndecan-4 is located at focal adhesions [163,167]. Its role in mechanotransduction is derived from experiments in cultured fibroblasts that were mechanically stressed. By using specific syndecan-4 antibodies it was shown that downstream intracellular signaling was increased with mechanical activation in cells attached solely through syndecan-4 [168]. It was also shown that presence of actin cytoskeletal filaments connected to focal adhesions was important for the syndecan-4-induced response to mechanical stress. Moreover, Herum et al. [169] showed that cardiac fibroblast expression of collagen I and III is dependent on syndecan-4, demonstrated using cardiac fibroblasts from mice lacking syndecan-4. In vivo experiments on these mice showed that molecular markers of myofibroblast differentiation were upregulated in wild-type mice, but not in mice lacking syndecan-4, following pressure overload. Thus, in vivo and in vitro experiments clearly indicate that syndecan-4 is part of a mechanosensory apparatus in cardiac fibroblasts, affecting myofibroblast transitioning and collagen expression. 

As indicated above, syndecan-4 induces fibroblast activation and differentiation through regulation of intracellular signaling complexes and pathways. Although details on how syndecan-4 transduces mechanical stress into intracellular signals are still to be elucidated, it has been convincingly shown that syndecan-4 is necessary for formation of focal adhesions [163,167], important sites for mechanotransduction, located at the termini of cytoskeletal actin stress fibers. Recently, it was shown that syndecan-4 was necessary for attachment of two important components of focal adhesions, vinculin and F-actin, to the cytoskeleton [170]. Specific intracellular signals involved in regulating syndecan-4 and its function are protein kinase C isoforms, phosphatidylinositol-(4,5)-bisphosphate and the Rho-family of GTPases [163,167]. It is currently a priority in our group to identify the nodal points in stress-induced syndecan-4 signaling and how it interacts with integrins to form focal adhesions, thereby regulating further downstream signals leading to activation of fibroblasts.

A family of signaling molecules known to induce activation of fibroblasts is the nuclear factor of activated T-cells (NFAT) family [153,154]. When active, the phosphatase calcineurin dephosphorylates NFATc1-4 initiating nuclear translocation and expression of genes associated with fibroblast differentiation. During increased cardiac and cellular stress, the calcineurin-NFAT pathway was activated by syndecan-4 [171]. In fibroblasts, nuclear translocation of the NFATc4 isoform occurred in response to cyclic mechanical stretch of fibroblasts in a calcineurin-dependent manner [169,172]. Blocking this pathway inhibited collagen production and myofibroblast differentiation [169]. Calcineurin is regulated by calcium, and it was recently demonstrated that syndecan-4 controls calcium homeostasis in focal adhesions. This is accomplished upon syndecan-4-ligand binding, which in turn phosphorylates transient receptor potential canonical 7 (TRPC) 7 channels thereby stabilizing it in its closed conformation [173]. It has also been shown that syndecan-4 mobilizes TRPC6 channels [174] and that TRPC6 is required for activation of calcineurin/NFAT signaling and myofibroblast activation in response to TGFβ [175]. Thus, emerging data indicate there is an important connection between syndecan-4 and TRPC-calcium in regulating calcineurin-NFAT signaling and thus, fibroblast activation and myofibroblast differentiation in response to mechanical stress.

In aortic stenosis patients and mouse models of myocardial infarction and pressure overload, a role for full-length syndecan-4 in stress-induced fibrosis has been shown [109]. Syndecan-4 is expressed in cardiac fibroblasts as well as myocytes and affect remodeling related to both cell types in the pressure-overloaded hearts of mice and men [111]. Experimental studies manipulating expression of syndecan-4 in vivo demonstrate its importance for stress-induced fibrosis. When pressure overload is induced in syndecan-4 knock-out mice, they do not develop concentric hypertrophy as wild-type mice [89,171], and importantly, myocardial stiffness is reduced owing to a reduced number of myofibroblasts and attenuated collagen cross-linking [89]. Yet, it remains to be explored whether targeting syndecan-4 and its associated pathways is a useful approach to prevent cardiac fibrosis and diastolic dysfunction.

Interestingly, not only the full-length syndecan-4 protein is upregulated in the mechanically stressed heart, but also the shedding of its extracellular domain is increased [111]. Shed syndecan-4 is found in myocardial tissue biopsies from heart failure patients and in the coronary sinus of patients with aortic stenosis, suggesting shedding from cardiac cells in response to persistent mechanical stress [111]. In cardiac fibroblasts in culture, expression levels and shedding is regulated by interleukin (IL) 1β, tumor necrosis factor (TNF) α and lipopolysaccharide (LPS)/TLR4 through a functional NFκB site in its promoter [111]. Several proteases shed syndecan-4 ectodomains in culture, although the relevant enzymes in the mechanically stressed heart remain to be elucidated.

Functionally, shedding of syndecan-4 in the heart represents an important aspect of syndecan-4 biology [109,163]. Cardiac fibroblasts in culture exposed to increased levels of shed syndecan-4 respond with altered expression of ECM genes [106], favoring ECM degradation. Cardiac fibroblasts treated with shed syndecan-4 fragments show reduced collagen I and III expression and reduced proliferation. Thus, the shed fragment has opposite effects on collagen expression compared to full-length syndecan-4. Accordingly, viral expression of the extracellular domain of syndecan-4 in the heart in vivo impaired cardiac function and infarct healing [176]. Thus, controlling syndecan-4 shedding, which may be possible by inhibiting receptors for IL-1β, TNFα or LPS, or through small molecules affecting central shedding enzymes, may affect cardiac fibrosis.

Syndecan-1, 2 and 3 are not located in focal adhesions and have so far not convincingly been shown to be involved in mechanotransduction. However, it has been shown that syndecan-1 is involved in cardiac fibrosis [177,178,179]. It is upregulated following myocardial infarction [166] and pressure overload [111], suggesting a role in the mechanical stress response of the heart. Although syndecan-1 may not be directly involved in mechanosensing, data clearly indicate that it is an essential mediator of angiotensin-II-induced cardiac fibrosis. Syndecan-1 knock-out mice display attenuated cardiac fibrosis upon angiotensin-II infusion. Moreover, it was shown that lack of syndecan-1 was associated with reduced expression of collagen I and III and of connective tissue growth factor (CTGF), downstream targets of TGFβ and YAP [180]. Thus, syndecan-1 might be involved in the mechano-regulation of fibrosis through cross-talk with these signaling pathways. Interestingly, it has been shown that circulating syndecan-1 correlates with markers of fibrosis and predicts clinical outcome in patients with HFpEF [181], and thus syndecan-1 is suggested as a blood biomarker of fibrosis that may add valuable diagnostic and prognostic information in HFpEF patients.

### 5.3. Stretch-Activated Ion Channels (SACs); Functional Roles in Fibrosis Still to Be Explored

Another means of cell surface mechanotransduction is ion currents through mechanosensitive channels. Although direct proof of mechanoactivation of these channels in cardiac fibroblasts is limited, we will briefly review the current knowledge of this mechanism in myofibroblasts across different organ systems that develop fibrosis, including heart, liver, lung and vasculature.

Cardiac fibroblasts are considered non-excitable cells, yet they do express several ion channels including voltage-gated sodium channel Na_v_1.5 [182], ATP-sensitive potassium channels (K_ATP_) [183], calcium- activated big potassium channels (BKCa) [184], and non-selective cation channels [175,185,186,187]. Mechanical cues can lead to opening of so-called stretch-activated ion channels (SACs). TRPCs are candidates for the stretch-activated currents measured in cardiac fibroblasts (Figure 3) [187,188,189]. In agreement, myofibroblast differentiation in response to TGFβ and matrix stiffness depends on the presence of TRPC3 [190], TRPC6 [175] and transient receptor potential vanilloid (TRPV) 4 [186,191] and involve Smad, MRTF-A and Akt signaling. Pharmacological inhibition or genetic ablation of TRPC3 and TRPV4 prevent fibrosis in pressure-overloaded hearts [190] and bleomycin-treated lungs [192], respectively, suggesting central roles for these channels in pro-fibrotic signaling.

Although not yet studied in the heart, the most solid data for a stretch-activated channel is Piezo1 [193] which is important for volume homeostasis in erythrocytes [194]. Interestingly, low levels of Piezo1 mRNA are detected in mouse heart, and Piezo1 channel electrophysiological properties are similar to that of endogenous cardiac cation non-selective stretch-activated channels. It will be exciting to follow the future elucidation of the role of Piezo1 in cardiac fibroblasts.

## 6. Mechanotransduction by the Cytoskeleton: Actin’ on Nuclear Translocation

The cytoskeleton is a dynamic structural network essential for cell shape, stability and function, and provides physical connectivity throughout the cell. The cytoskeletal pre-stress (the level of isometric tension in the cytoskeleton), is constantly adjusted to create a balance between cytoskeletal stiffness and ECM stiffness. Thus, ECM stiffness is a crucial regulator of cytoskeletal pre-stress [43,195] which is largely determined by the formation of polymeric F-actin fibers from G-actin monomers.

The mechanisms whereby cells adjust pre-stress involve mechanotransduction signaling pathways from surface molecules (e.g., integrins and syndecans) to the family of small Rho GTPases, of which RhoA has been extensively studied [196]. RhoA interacts with, and thereby activates, Rho kinase (ROCK) which phosphorylates several downstream targets involved in stress fiber formation and dissociation, including LIM kinases (LIMK) and myosin light chain (MLC). LIMK will phosphorylate cofilin, an actin depolymerizing factor which is inhibited by phosphorylation [197], while phosphorylation of MLC directly increases actin contractility. The net result of RhoA activity is enhanced actin fiber formation [196] which subsequently affects nuclear translocation of transcription factors and thereby gene regulation.

### 6.1. Myocardin-Related Transcription Factors Are Liberated from Actin by Mechanical Stimulation

The myocardin-related transcription factors (MRTF) A and B bind to G-actin monomers present in the cytosol under low tension conditions. In response to enhanced extracellular mechancal cues, G-actin assembles into F-actin polymers thereby liberating MRTF from G-actin and allowing it to enter the nucleus (Figure 3) [198,199,200]. Interestingly, nuclear shuttling of MRTF seems also to be controlled by nuclear RhoA activity and its effector mDia2, which can shuttle between the nucleus and cytoplasm. Nuclear actin polymerization, Rho and the mammalian homolog of Diaphanous (mDia) all activate MRTF [201] which initiates gene transcription by acting as a cofactor for serum response factor (SRF). SRF binds to CArG box elements in the promoter region of its target genes which comprise a group of smooth muscle (and myofibroblast) marker genes including SMA and SM22. Genetic knockout of MRTF-A reduces fibrosis and scar formation following myocardial infarction [202] and in bleomycin-induced pulmonary fibrosis [203]. Thus, MRTF-A is essential for myofibroblast differentiation of development of cardiac fibrosis in response to mechanical cues.

### 6.2. Hippo Signaling Pathway Is Regulated by Cytoskeletal Dynamics

The Hippo signaling pathway has emerged as an important mechanotransduction pathway in response to matrix stiffness [204,205,206]. Although the identity of the mechanosensor that initiates Hippo signaling is not clear, cytoskeletal dynamics are central for pathway activity. Thus, inhibition of Rho and disruption of F-actin results in pathway inactivation [207]. When Hippo signaling is absent, the transcription coactivators YAP and its paralog TAZ (transcriptional coactivator with PDZ-binding motif) can translocate between the cytoplasm and nucleus. YAP and TAZ are phosphorylated by upstream kinases which, depending on the phosphorylation site, target them for degradation [208] or retain them in the cytoplasm [209] through binding to cytoplasmic angiomotin. F-actin regulates YAP activity by competitive binding to angiomotin thereby releasing YAP and allowing nuclear translocation [209,210]. Since F-actin is regulated by the mechanical properties of the ECM, a stiff matrix will promote nuclear translocation of YAP/TAZ while soft culturing conditions render them cytoplasmic [35,211].

Although YAP and TAZ have not yet been studied in cardiac fibroblasts, nuclear localization induces myofibroblast differentiation in liver [35,212], lung [211] and skin [213]. Mice lacking or overexpressing constituents of the Hippo signaling pathway have demonstrated reduced and increased fibrosis, respectively [214,215]. Likewise, knockdown or pharmacological inhibition of YAP/TAZ delays wound healing [216] and prevents TGFβ-induced renal fibrosis [217]. Indeed, upregulation of ECM genes, including CTGF has been found to depend on YAP/TAZ activity [180].

Since YAP and TAZ are transcriptional co-activators, they regulate gene expression through interaction with transcription factors, including those of the TEA domain (TEAD) transcription factor family [218]. Interestingly, direct interaction of YAP/TAZ with Smad3 of the TGFβ signaling pathway has been shown in keratinocytes [219] and LLC-PK1 cells [220]. As such, YAP/TAZ activation may comprise a mechanism for the established necessity of mechanical stress for TGFβ-induced myofibroblast differentiation [221]. However, YAP/TAZ do not require TGFβ to induce pro-fibrotic effects in response to matrix stiffness [35,211]. In line with this, TAZ was recently found to enhance Smad3 sensitivety to the Acta2 (SMA) promoter, synergize with MRTF in binding to TEAD elements, while antagonizing MRTF binding to the Acta2 promoter [220]. Thus TAZ seems to facilitate mechanical and/or chemical pro-fibrotic signaling depending on the context.

Collectively, these results demonstrate the importance of the cytoskeleton in regulating the pro-fibrotic gene program by acting on nuclear translocation of transcription regulators.

## 7. Nuclear Mechanosensing: Long-Distance Communication

The cytoskeleton provides a physical coupling from cell surface focal adhesions to protein complexes in the nuclear membrane. This extraordinary structural connectivity throughout the cell enables long-range force propagation from ECM structures directly to the nucleus thus allowing propagation of mechanical signals more rapidly than by chemical diffusion or translocation-based signaling [222].

The nucleus consists of the nuclear interior and the nuclear envelope, a continuous membrane system comprising the outer and inner phospholipid bilayer membranes and the nuclear lamina on the interior side of the membrane. The translation of mechanical cues into changes in cell phenotype is accomplished by specialized proteins that are part of the linker of the nucleoskeleton and cytoskeleton (LINC) complex that span the nuclear envelope bridging the cytoskeleton to the nuclear lamina (Figure 3) [223]. This complex regulates transcription factors and chromatin structure in the nucleus and thereby gene transcription [222,224]. Although studies examining cardiac fibroblasts are sparse, data from fibroblast cell lines and mouse embryonic fibroblasts shed light on nuclear mechanotransduction events most likely also relevant to cardiac fibroblasts.

### 7.1. Linker of Nucleoskeleton and Cytoskeleton; A Complex for Nuclear Detection of Force

Starting from the cytosolic side, the LINC complex consists of transmembrane nesprin proteins (nuclear envelope spectrin repeat protein) [225] that connect the cytoskeleton to the outer nuclear membrane through binding of actin (for nesprin-1 and -2) and the intermediate filament binding protein plectin (for nesprin-3) at their N-terminal [226,227]. Their C-terminal KASH (Klarsicht, ANC-1, Syne Homology) domain is connected to the C-terminal Sad1-UNC (SUN) homology domains of SUN proteins that protrude from the nuclear lumen into the perinuclear space between the inner and outer nuclear membranes [228,229]. Importantly, SUN1 binds directly to lamin A [230] and B [231] of the nuclear lamina thereby anchoring the LINC complex to the nucleoskeleton. Lamins are specialized fibrous proteins that form the nuclear lamina and provide structural stability to the nucleus. Other integral inner membrane proteins include emerin, MAN1, lamina-associated polypeptide-emerin-MAN1 (LEM) 2, Samp1 and barrier-to-autointegration factor (BAF) which are involved in nuclear organization and bind to lamin, DNA and various transcription factors [232,233]. 

Gene mutations, gene knockout and dominant negative expression of LINC complex proteins have provided insight to their functions in mechanotransduction [234,235]. Mutations in the LMNA gene encoding lamin A and C (resulting from alternative splicing), emerin and nesprins cause diseases called laminopathies. The effects of these mutations are particularly apparent in tissues that are under constant mechanical load, such as skeletal and cardiac muscle, resulting in muscular dystrophy and dilated cardiomyopathy, respectively [236]. Fibroblasts from Lmna knockout mice show altered proliferation, thus lamin A/C is likely important for fibroblast activation and possibly differentiation into myofibroblasts [237]. In agreement, lamin A/C and emerin have been found to modulate actin dynamics in mouse embryonic fibroblasts, regulating MRTF-A and SRF activity [238] which is essential for myofibroblast differentiation in the heart [202].

Direct detection of force transduction across the LINC complex was recently accomplished using a nesprin-2G FRET-based tension biosensor [239]. The force sensitivity of this biosensor is dependent on actomyosin tension and cell shape, and LINC tension was reduced in fibroblasts from patients with the LMNA genetic disorder Hutchinson-Gilford progeria syndrome with abnormal nucleus shape [239].

The mechanism whereby the LINC complex translates mechanical cues into gene regulatory events, is poorly understood. However, there is evidence that direct interaction with transcription factors may take place. Another possible mechanism is modification of nuclear import of gene regulatory proteins [240] and export of mRNA through the nuclear pore complexes [241]. Of relevance for fibrosis, TGFβ-induced nuclear translocation of the transcription factor Smad3 was hampered in fibroblasts from nesprin-2 knockout mice [240].

The short isoform of transcriptional elongation factor bromodomain-containing protein 4 (Brd4) is localized to the inner nuclear membrane where it associates with SUN proteins of the LINC complex and binds to acetylated histones [242]. Brd4 expression levels correlate with ECM gene expression and Brd4 is involved in gene regulation through RNA processing and chromatin modifications. Brd4 was recently identified as a critical regulator of lung and liver fibrosis [243,244] and blocking Brd4 not only prevents but also reverses fibrosis and myofibroblast differentiation of hepatic stellate cells [244]. Interestingly, Brd4 inhibition also reduces myocardial damage following infarction [245] suggesting a role for Brd4 in the heart.

Finally there is evidence that the LINC complex regulates gene expression by modifying gene accessibility and chromatin packaging. Indeed, SUN proteins bind directly to chromatin and are central for chromosome organization during mitosis, reflected in SUN1 knockout mice being infertile due to the inability to implement cell division [246]. However, the mechanism could also be of more indirect character, regulating gene expression as a result of altered nuclear structure, shape and/or stiffness. Indeed, substrate stiffness has been found to be important for LINC complex assembly [247].

### 7.2. Nuclear Shape and Stiffness Is Associated with Cardiac Fibrosis

The nucleus itself has a stiffness in the range of 0.1 to 10 kPa [248] which is 2–10 times higher than the cytoskeleton [249,250]. Changes in nuclear shape and stiffness are associated with changes in gene expression and cell differentiation status. Thus it is likely that this physical parameter may play a role in cardiac fibroblast differentiation into myofibroblasts. Indeed, there are strong correlations between changes in nuclear shape and changes in fibroblast collagen synthesis in osteogenic cells [251]. Conversely, nuclear shape is affected by substrate stiffness as a result of stiffness-induced changes in actomyosin tension being more round on soft substrates (0.4 kPa) and flattened on stiff substrates (308 kPa) [252]. Nuclear shape was associated with cell responses to substrate stiffness including cell spreading and migration speed. This “stiffness-sensing” was lost when the LINC complex was disrupted suggesting that an intact nucleus-cytoskeleton connection is required for rigidity sensing. 

The amount and localization of densely packed heterochromatin has been found to affect the physical properties of the nucleus, providing structural stability and rigidity when located in the nuclear periphery in proximity of the nuclear lamina. Likewise, the location of chromatin affects packaging and thereby gene transcription, being more actively transcribed when located in the center of the nucleus and silenced along the periphery [253]. Recently it was demonstrated that physical deformation of the nucleus can rapidly (less than 15 s) modulate gene expression [254] by directly affecting chromatin structure. It is likely that this is a ubiquitous and efficient way for cells to accommodate transcriptional activity in response to external mechanical forces.

Lamins are essential for nuclear lamina structure and thereby nuclear stiffness. Cardiac cells from Lmnaknock-out mice have disrupted nuclear lamina. This is also observed in mice overexpressing the nucleosomal binding protein high mobility group nucleosome binding domain 5 (HMGN5) [255]. HMGN5 disrupts H1 histone and chromatin interaction and thereby reduces chromatin compaction [256], suggesting a link between lamin and chromatin structure. Similar to Lmna knock-out mice, HMGN5 overexpressing mice die before 8 weeks of age due to heart failure characterized by ventricular wall thinning and substantial fibrosis [255]. Nuclei of cardiac fibroblasts and myocytes in the maturing heart are exposed to increasing mechanical stress. Thus, the fragile nucleus with reduced stiffness displays decompacted chromatin and nuclear blebbing, characteristics of cell death. These observations emphasize the mechanosensitivity of nuclei and the importance of nuclear structural integrity for cardiac cell adaption to mechanical forces.

Several questions remain regarding the detailed mechanisms of this fascinating long-range communication system that enables cells to sense and translate extracellular mechanical cues into transcriptional changes. Together with advanced molecular biology methods to study chromatin dynamics and the rapidly growing knowledge of epigenetic gene regulatory mechanisms, a better understanding of nuclear mechanotransduction, also in cardiac fibroblasts, is expected in the near future. 

## 8. Manipulating the Soft and Hard-Heartedness of Cardiac Fibroblasts

The consequences of cardiac fibrosis are widespread and detrimental and despite promising results in animal models, clinical trials of anti-fibrotic therapies have been disappointing [11,12,13,14,15,257]. Drugs targeting the renin-angiotensin-aldosterone system, endothelin, inflammatory cytokines and TGFβ have shown either modest regression of fibrosis, or adverse effects on the vasculature and liver [257]. A beneficial effect of short-term infusion of the endogenous hormone relaxin in acute heart failure has shown promise [258] and anti-fibrotic effects are clear in animal models of cardiac disease. Here the challenge lies in the short half-life and costly production of this hormone. Furthermore, most tested drugs are administered orally or through injections, i.e., are systemically administered, increasing the chance of non-cardiac effects and hampering obtainment of sufficiently high cardiac dosages without having toxic effects on other organs. These obstacles could potentially be overcome by localized delivery or cardiac-specific targeting of future anti-fibrotic treatments.

A more specific targeting of cardiac TGFβ might involve blocking mechanical activation of latent TGFβ. This could potentially be accomplished by inhibiting αv integrin binding [60,70,187,259], or inhibiting myofibroblast contraction using peptides that prevent the incorporation of SMA into stress fibers [260]. For such an approach to be successful, directed targeting of cardiac myofibroblasts is crucial since adhesion through integrins, and SMA fiber formation and contraction is an essential part of normal smooth muscle cell function throughout the body.

Targeting mechanotransduction by the cytoskeleton also holds potential. Novel small molecule inhibitors of Rho/MRTF-A signaling have been shown to prevent bleomycin-induced dermal fibrosis in mice by oral administration, prevent scar tissue formation in a preclinical model of conjunctival fibrosis [261] and prevent pulmonary fibrosis in two distinct mouse models [262]. Further optimization of these compounds’ stability, solubility and potency has made them suitable for long-term treatment of fibrosis. It will be exciting to see whether these will succeed in clinical trials and whether they could be effective for treatment of fibrosis in the heart.

Verteporfin, a drug used to treat certain types of eye diseases, was recently found to inhibit YAP [263] and dramatically reduce YAP/TAZ protein levels thereby preventing TGFβ-induced renal fibrosis through smad2/3 [217]. Although the anti-fibrotic effect of verteporfin needs to be examined in the heart, there might be therapeutic potential in repurposing this clinically approved and used drug.

One particular challenge using pharmacological inhibitors of mechanotransduction in the heart, as described above, is the importance of these same pathways in cardiomyocytes. Thus, inhibition will not only affect fibrosis, but likely also cardiomyocyte function and hypertrophic remodeling [264,265]. This is not necessarily a disadvantage, as balanced hypertrophic remodeling and fibrosis is key to cardiac function, but it nevertheless represents a challenge when pursuing such therapeutic avenues.

Pharmacological treatments do not primarily interfere with the physical properties of the extracellular matrix, which this review clearly emphasizes are crucial for cardiac fibroblast function and phenotype. The increased matrix stiffness that accompanies fibrosis will favor the persistent presence of pro-fibrotic activated cardiac fibroblasts, while a healthy cardiac fibroblast requires a stiffness corresponding to that of the healthy heart [30,266]. Cumulating data suggest that the myofibroblast phenotype is reversible [37,57,267,268,269,270], and that manipulating the stiffness of cell culturing conditions is sufficient to induce myofibroblast reversion [37]. Thus, an alternative approach to specific pharmacological targeting is the general targeting of mechanotransduction pathways by manipulating tissue stiffness in vivo thereby promoting re-establishment of a healthy cardiac fibroblast population in the fibrotic heart.

Recent advances in regenerative medicine and bioengineering has led to the development of biomaterials of varying stiffness with clinical translational potential [271,272,273,274]. These include synthetic hydrogels and naturally-derived matrices including decellularized cardiac porcine ECM [275] that can be injected into wounded tissues to reduce wall stress and provide the tissue with a matrix stiffness that promotes tissue repair. Indeed, following myocardial infarction in rats, injection of hydrogels into the infarct region in the beginning of the fibrotic phase led to improved cardiomyocyte survival and function, as well as reduced fibrosis both in the infarct and remote regions of the myocardium [272,276].

Whether a change in stiffness will alone be sufficient to reverse the cardiac myofibroblast phenotype in vivo is yet to be studied. It may be that a combination of pharmacological and mechanical approaches is needed. Indeed, anti-fibrotic drugs can be encapsulated in injectable hydrogels that gradually release the drug upon hydrogel degradation enabling local and sustained delivery of anti-fibrotic agents. This was recently demonstrated for an anti-fibrotic hepatic growth factor fragment that reduced fibrosis following myocardial infarction in rats [277]. Moreover, drugs targeting the mechanical memory of myofibroblasts may be useful in combination with injectable “soft” hydrogels. It was recently shown that microRNA-21 is responsible for sustainment of a mechanical memory in mesenchymal stem cells, and knockdown thereof promoted reversion of the pro-fibrotic phenotype when used in combination with culturing on soft substrates [270].

Injectable synthetic or native matrix-derived hydrogels are clinically attractive because of their minimal invasiveness as they can be delivered to the heart as liquid through intramyocardial catheters where after they polymerize into gels in the tissue. However, there are limitations and challenges to this approach that need to be addressed. Importantly, safety in human patients has to be assessed for translation from pre-clinical models. Although synthetic biomatrices are designed to mimic the properties of the natural tissue, differences in structure and composition to the native ECM exist, and native-derived hydrogels have been obtained from decellularized ECM from pigs, opening for challenges related to species-differences

Even though successful treatment of fibrosis is currently lacking, there have been enormous advances in this field over recent years providing substantially more insight into cardiac fibroblast physiology and pathophysiology and opening for translation of pre-clinical findings that in the future may offer benefit for patients. A better understanding of the importance of mechanical cues in regulating the pro-fibrotic signaling in the heart is part of this foundation. It is likely that this increased knowledge will enable manipulation and control of the soft- and hard-heartedness of cardiac fibroblasts and hopefully, ultimately enable the prevention, reduction and reversion of cardiac fibrosis.
